# Differential Effects of Poor Recall and Memory Disjointedness on Trauma Symptoms

**DOI:** 10.1177/2167702619847195

**Published:** 2019-05-23

**Authors:** Juliane Sachschal, Elizabeth Woodward, Julia M. Wichelmann, Katharina Haag, Anke Ehlers

**Affiliations:** 1 Centre for Anxiety Disorders and Trauma, Department of Experimental Psychology, University of Oxford; 2 Oxford Health National Health Service Foundation Trust

**Keywords:** PTSD, trauma, memory, intrusions, cognitive processing

## Abstract

Clinical theories of posttraumatic stress disorder (PTSD) suggest that trauma memories are disorganized. In the present study, we examined how trauma-film exposure affects two aspects of memory disorganization, poor memory recall and memory disjointedness, and their relationship to PTSD-like symptoms. In Session 1, 90 healthy participants were exposed to a trauma (*n* = 60) or a neutral film (*n* = 30). Cognitive processing styles, memory characteristics, and intrusive memories of the film were assessed. The trauma-film group reported greater memory disjointedness of the worst moments of the film but better memory recall of the film than the neutral-film group. In the trauma-film group, cognitive processing and memory disjointedness were related to intrusive memories and PTSD-like symptoms in the week after film exposure. Memory disjointedness but not poor memory recall mediated the relationship between cognitive processing and intrusions. The findings suggest that different aspects of memory disorganization need to be distinguished to explain PTSD symptoms.

Trauma survivors with posttraumatic stress disorder (PTSD) involuntarily re-experience facets of their trauma very vividly, while at the same time experiencing difficulties in voluntarily recalling some aspects of the trauma (American Psychiatric Association, 2013). Cognitive theories of PTSD account for this phenomenological paradox by suggesting that predominantly perceptual cognitive processing (data-driven processing, dissociation) during trauma leads to disorganized trauma memories and a lack of integration into their context (e.g., Brewin, Gregory, Lipton, & Burgess, 2010; Ehlers & Clark, 2000). This would suggest that people with PTSD have more disorganized trauma memories compared with trauma survivors without PTSD and compared to negative but nontraumatic control events. Other authors have argued that trauma memories do not differ from other important memories (Berntsen, Willert, & Rubin, 2003; Rubin, Berntsen, & Bohni, 2008) and that the same processes that contribute to trauma memories in PTSD should be relevant for memories of highly negative events in general. Furthermore, they argued that incoherence of trauma memories in PTSD can be accounted for by cognitive impairments that are common in people with PTSD (Rubin et al., 2016).

To date, the literature on memory disorganization in PTSD is inconclusive. Brewin (2016) pointed out that discrepancies in the recent literature on trauma memories can partly be explained by differences in the type of narrative and focus of analysis, suggesting that a refinement in the analysis of trauma-memory impairment in PTSD is warranted. Furthermore, the analysis of memory for naturally occurring trauma involves the difficulty that it is unknown to the investigators what happened in the trauma. Experimental induction of an analogue trauma with the trauma-film paradigm might help to better understand current discrepancies in the literature on memory disorganization. In these studies, healthy participants are exposed to film material involving serious harm to other people (e.g., a rape or severe accident); cognitive processing during the film, memory for aspects of the film, and subsequent intrusive memories are assessed. Several studies have examined peritraumatic processing and the development of PTSD-like symptoms with trauma-film exposure (e.g., Halligan, Clark, & Ehlers, 2002; Holmes, Holloway, Brewin, & Hennessy, 2004) and found that the qualities of reported intrusive memories were similar to those reported by people with PTSD (e.g., Weidmann, Conradi, Gröger, Fehm, & Fydrich, 2009). The trauma-film paradigm thus seems suitable to examine memory qualities after analogue trauma to further the understanding of the development of re-experiencing symptoms and to complement the studies of people after real-life trauma. Overall, the literature on memory qualities after exposure to real-life trauma or trauma films has so far shown some support for the role of cognitive processing styles and self-reported memory disorganization in PTSD symptoms, but there are also some divergent findings.

## Prediction of intrusive memories

Prospective studies of trauma survivors showed that self-reported memory disorganization (e.g., Ehring, Ehlers, Cleare, & Glucksman, 2008; Halligan, Michael, Clark, & Ehlers, 2003; Murray et al., 2002) predicted the development of PTSD symptoms after trauma. They also showed that self-reported dissociation and data-driven processing during trauma predicted the development of PTSD (e.g., Ehring et al., 2008; Halligan et al., 2003; Murray et al., 2002). Trauma-film studies found that subjective (Halligan et al., 2002, Study 2; Kindt, Van Den Hout, & Buck, 2005) but not objective (Kindt & Van Den Hout, 2003; Kindt et al., 2005) measures of memory disorganization predicted PTSD symptoms after analogue trauma and that dissociation (e.g., Kindt et al., 2005, Study 2; Sachschal, Suendermann & Ehlers, in prep.) and data-driven processing (e.g., Halligan et al., 2002: Study 2) were associated with re-experiencing symptoms after analogue trauma.

## Trauma narratives

Many studies of trauma survivors have investigated whether the overall trauma narrative is disorganized in PTSD (e.g., Foa, Molnar, & Cashman, 1995), manifesting in difficulties in memory recall (e.g., in accessing details of what happened), the order of events during the trauma, or incoherent accounts of the trauma. Most studies using objective ratings of trauma narratives found that trauma memories were more disorganized in people with PTSD or acute stress disorder compared with healthy control subjects (De Young, Kenardy, & Spence, 2007; Halligan et al., 2003; Harvey & Bryant, 1999; Jelinek, Randjbar, Seifert, Kellner, & Moritz, 2009; Jones, Harvey, & Brewin, 2007; Salmond et al., 2011). Results for self-report measures are less clear. Although the majority of studies found greater self-reported memory disorganization for trauma survivors with PTSD than traumatized control subjects (Halligan et al., 2003; Jelinek et al., 2009), one study did not (Berntsen et al., 2003).

## Specificity

It remains unclear whether overall memory disorganization is specific for trauma memories in PTSD or whether PTSD is associated with a more general impairment in autobiographical memory. To address this, several studies also compared the characteristics of trauma memories in PTSD to those of negative control events. Some studies found greater memory disorganization for traumatic compared with negative control events in PTSD with self-report measures (Ehlers et al.,2019; Halligan et al., 2003) or independent ratings of narratives (Jelinek et al., 2009; Salmond et al., 2011). However, other studies did not find specificity in self-report measures (Jelinek et al., 2009; Megías, Ryan, Vaquero, & Frese, 2007; Rubin, Boals, & Berntsen, 2008) or objective ratings of trauma narratives (Rubin, 2011; Rubin et al., 2016).

The inconsistent results may be due in part to the fact that some of the operationalizations of memory disorganization, such as difficulties with recalling aspects of the event, also apply to autobiographical memories in general (Berntsen et al., 2003; Rubin, Berntsen, et al., 2008). In an attempt to specify the critical features of memory disorganization in PTSD more precisely, Ehlers, Hackmann, and Michael (2004) proposed that the subjectively worst moments of the trauma are disjointed from relevant context information in memory (e.g., what happened before or afterward). Two studies found that narratives of the moments of the trauma memory that matched the content of intrusive re-experiencing were more disorganized compared with other moments of the trauma narrative that were not re-experienced (Evans, Ehlers, Mezey, & Clark, 2007; Jelinek et al., 2010). Kleim, Wallott, and Ehlers (2008) further found that compared with trauma survivors without PTSD, people with PTSD took longer to access other information from autobiographical memory while listening to the worst parts of the trauma.

To date, it has not been examined how trauma exposure affects different aspects of memory disorganization and which aspects of memory disorganization are most relevant to the development of PTSD symptoms. In the present study, we therefore used a trauma-film paradigm to investigate (a) whether trauma exposure differentially affects two aspects of memory disorganization, namely difficulties in recall and disjointedness; (b) which of these better predict intrusions; and (c) which aspect of memory disorganization can account for the relationship between peritraumatic cognitive processing and the development of PTSD symptoms. We hypothesized that (a) trauma-film exposure leads to difficulties in memory recall and greater memory disjointedness of the worst moments compared with neutral-film exposure; (b) disjointedness shows greater differences than difficulties in recall; (c) in the trauma-film group, difficulties in memory recall, memory disjointedness, and peritraumatic cognitive processing predict the development of intrusions; and (d) difficulties in memory recall and memory disjointedness mediate the relationship between peritraumatic processing and the development of intrusions after trauma-film exposure.

## Method

### Participants

Ethical approval was obtained from the Medical Sciences Inter-Divisional Research Ethics Committee of the University of Oxford. Ninety participants (61 female, 28 male, 1 gender not specified) between 18 and 58 years (*M* = 24.08; *SD* = 5.70) were recruited via online and poster advertisements and circular emails to students and staff of the University of Oxford. Inclusion criteria were healthy participants aged between 18 and 65 years. The exclusion criterion was a history of an interpersonal trauma. Participants were randomly allocated with a 2:1 allocation schedule, stratified by gender, to either exposure to a trauma film (*n* = 60) or to a neutral control film (*n* = 30). Participants in the trauma-film group had a mean age of 24.05 years (*SD* = 5.68) and a mean of 16.65 years of education (*SD* = 3.55). In the neutral-film group, they had a mean age of 23.86 years (*SD* = 5.52) and a mean of 17.31 years of education (*SD* = 2.93). Socioeconomic background was not assessed. All 90 participants (100%) attended the 1-week follow-up and 75 (50 from the trauma-film group, 25 from the neutral-film group) completed an online questionnaire at Day 3 (83%).

### Film material

In the trauma-film condition, participants saw a 10.5 min clip from the film *Irréversible* (Noé & Rossignon, 2002), in which a young woman walks home at night and is raped by a stranger. In the neutral-film condition, participants saw a neutral YouTube clip, in which a man and a woman talk about language differences in Quebec, that was matched with the trauma film for color, duration, and number of actors (one man, one woman). Participants wore headphones and watched the clips on an iMac in full screen mode and were situated approximately 60 cm away from the screen.

### Measures

#### General Information Questionnaire

This questionnaire gathers information about the participants’ demographic characteristics and their education.

#### State Dissociation Questionnaire

State dissociation was assessed with the 9-item State Dissociation Questionnaire (SDQ; e.g., “I felt distant from my emotions”; Murray et al., 2002). Participants rated how much each statement applied to them on a scale from 0 (*not at all*) to 4 (*very much so*). Mean scores are reported. Internal consistency (Cronbach’s α) was .85.

#### Data-Driven Processing Scale

Data-driven processing was measured with the 8-item Data-Driven Processing Scale (DDPS; e.g., “It was like a stream of unconnected impressions following each other”; Halligan et al., 2002). Participants rated how much each statement applied to them on a scale from 0 (*not at all*) to 4 (*very much so*). Mean scores are reported. Internal consistency (Cronbach’s α) was .76.

#### Subjective Units of Distress Scale

Subjective distress was assessed with an adapted version of the Subjective Units of Distress Scale (SUDS; Wolpe, 1958). Participants rated their distress on a scale from 0 (*no distress, totally relaxed*) to 100 (*highest anxiety/distress that you have ever felt*).

#### Negative Event Memory Questionnaire (MQ)

The Memory Questionnaire, developed by Halligan et al. (2003), was adapted to more clearly distinguish between disjointedness, that is, poor links between different parts of the memory and preceding and subsequent information (four items; e.g., “My memories of the worst moments of the film feel disconnected from/not joined up with/separate from what happened beforehand and afterwards”), and aspects of difficulties in recall, for example, memory gaps or difficulty remembering the order of the event (four items; e.g., “I feel that my memory for the film is incomplete”). Participants rated how much each statement applied to their memory of the film on a scale from 0 (*not at all*) to 4 (*very much so strongly*). Table 1 displays the items of both scales. A previous version of the memory-disorganization scale has been found to predict PTSD-like symptoms after an analogue trauma film (Halligan et al., 2002). Internal consistency (Cronbach’s α) was .74/.79 for the poor memory recall/disjointedness scales at Day 3 and .84/.74 at 1-week follow-up.

**Table 1.** Items of the Memory Questionnaire for Poor Memory Recall and Memory Disjointedness Subscales

Item no.	Item
Poor memory recall	
1	I feel that my memory for the film is incomplete.
2	I have trouble remembering the order in which things happened during the film.
3	My memory for the film is muddled.
4	I cannot get what happened during the film straight in my mind.
Memory disjointedness	
5	I remember different parts of the film like separate scenes.
6	When I remember a particular upsetting part of the film, it is hard to remember that it was a film.
7	My memories of the worst moments of the film feel disconnected from / not joined up with / separate from what happened beforehand and afterwards.
8	Some moments of the film come back into my mind unchanged, just as they were right after seeing the film.

#### Intrusion diary

Intrusions during the week were assessed with an online daily diary designed using Qualtrics software (Version 01/2016; Qualtrics, Provo, UT). Participants were sent daily email reminders to fill in the diary in the morning and evening of each day. Participants were asked to report any unwanted intrusions of images of the film that they experienced during the day.

#### Intrusion interview

The intrusion interview assessed visual intrusions over the last 7 days. The interview assessed the content, frequency, and persistence of unwanted images of the film.

#### Impact of Event Scale-Revised

The 33-item Impact of Event Scale–Revised (IES-R; Horowitz, Wilner, & Alvarez, 1979) measures intrusions (e.g., “I thought about it even if I did not mean to”), avoidance (e.g., “I stayed away from reminders of it”), and arousal symptoms (e.g., “I felt irritable and angry”). Participants rated how much they were bothered or distressed by the difficulties described in each item in the last 7 days on a scale from 0 (*not at all*) to 4 (*extremely*). Wording was adapted to be suitable for exposure to the film clips; for example, the word *trauma* was changed to *film*. Internal consistency was Cronbach’s α = .93.

### Procedure

Participants responded to circulars and advertisements about the study and were invited for two research sessions at the Department of Experimental Psychology at the University of Oxford and sent an information sheet. Session 1 took about 1 hr and Session 2 about 30 min to complete. On arrival at Session 1, participants were informed about the nature and procedure of the study and the experimenter ascertained that they met inclusion criteria. If this was the case, they gave written informed consent. Participants were then randomly assigned and exposed to either the trauma film clip or the neutral film clip. They were reminded that they could stop the film at any time. Afterward, they answered some manipulation-check questions and completed questionnaires about their responses to the film (SDQ, DDPS, SUDS). The experimenter made sure that participants were feeling all right before they went home. In the week after the session, participants were asked to complete the online daily intrusion diary. At Day 3, participants were asked to complete the MQ online from home. At Session 2 (1-week follow-up), participants completed the MQ, IES-R, and intrusion interview. Participants were reimbursed £30 for their time and travel expenses.

### Data analysis

Results were calculated with IBM SPSS Statistics (Version 24). Significance levels were set at α = .05, two-tailed. The PTSD (intrusions, IES-R), memory characteristic (poor recall, disjointedness), and peritraumatic processing (data-driven processing, dissociation) variables were skewed and log-transformed into normal (skewness values between –0.70 and 0.70; kurtosis values between –1.61 and 0.28). To test whether the manipulation had worked, the trauma- and neutral-film groups were compared with independent *t* tests (for distress, state dissociation, and data-driven processing during the film and intrusions and PTSD symptoms in the week after the film). To test Hypotheses 1 (greater difficulties in recall and disjointedness in the trauma-film group) and 2 (greater differences for disjointedness than for difficulties in recall), mixed-measures analyses of variance (ANOVAs) compared MQ scores with the between-subject factor group (trauma film, neutral film) and the within-subject factors time (Day 3, 1-week follow-up) and memory quality (disjointedness, difficulties in recall). To test Hypothesis 3 (both aspects of memory disorganization are related to intrusions and PTSD symptoms), Pearson correlations were calculated between difficulties in memory recall and memory disjointedness at Day 3 and 1-week follow-up and PTSD symptoms in the week after the film (intrusion diary, intrusion interview, IES-R). To test Hypothesis 4, mediation models were calculated using the process macro for SPSS (Hayes, 2017). Separate analyses were calculated using cognitive processing variables (dissociation, data-driven processing) as predictor variables *X*, memory characteristics as mediator *M*, and intrusions (diary, interview) or PTSD symptoms (IES-R) as outcome variable *Y*. Direct and indirect effects were calculated using bootstrapping approximation with 5,000 samples and a 95% confidence interval (CI).

## Results

### Responses to films and development of PTSD-like symptoms

Table 2 displays responses to the film clip, distress, and intrusion scores in the trauma-film and neutral-film groups. After film exposure, the trauma-film group reported more distress, *t*(88) = 13.90, *p* < .001, *d* = 3.11, 95% CI = [2.46, 3.73], data-driven processing, *t*(88) = −6.72, *p* < .001, *d* = 1.50, 95% CI = [1.01, 1.98], and state dissociation during the film, *t*(88) = −5.83, *p* < .001, *d* = 1.30, 95% CI = [0.82, 1. 87] compared with the neutral-film group. The trauma-film group also reported more intrusions at 1-week follow-up in the intrusion interview, *t*(88) = −7.94, *p* < .001, *d* = 1.76, 95% CI = [1.26, 2.28], the intrusion diary, *t*(88) = −7.96, *p* < .001, *d* = 1.78, 95% CI = [1.27, 2.29], and higher scores on the IES-R, *t*(88) = −15.61, *p* < .001, *d* = 3.49, 95% CI = [2.80, 4.16].

**Table 2.** Demographics, Responses During the Film and Development of PTSD-Like Symptoms, and Mean Scores for Self-Reported Memory Characteristics in Trauma-Film and Neutral-Film Groups on Day 3 and at 1-Week Follow-Up

	Trauma film (*n* = 60)		Neutral film (*n* = 30)	
Variable	*M*	*SD*	*M*	*SD*
Manipulation check				
Distress during film	70.08	27.25	0.67	1.56
Data-driven processing during film	1.13	0.63	0.39	0.30
Dissociation during film	0.61	0.62	0.07	0.15
Intrusions in week after film				
Diary	5.00	7.11	0.03	0.18
Interview	6.58	7.99	0.20	0.55
PTSD symptoms in week after film (IES-R)	20.18	11.46	0.90	1.45
Memory quality				
Disjointedness Day 3 (*n* = 75) 1-week follow-up Sample in ANOVA (*n* = 75) Total sample (*N* = 90)	2.88 3.50 3.55	3.25 3.58 3.51	0.84 0.96 0.90	1.31 1.72 1.60
Poor recall Day 3 (*n* = 75) 1-week follow-up Sample in ANOVA (*n* = 75) Total sample (*N* = 90)	1.30 2.12 2.02	1.88 2.70 2.66	2.56 4.16 4.07	3.90 4.31 4.14

Note: IES-R = Impact of Event Scale–Revised; ANOVA = analysis of variance. Scores at 1-week follow-up are displayed for the sample that completed Day 3 measures (*n* = 75) and were used in the ANOVA and the total sample (*N* = 90).

### Hypotheses 1 and 2: memory disjointedness and poor recall after trauma exposure

Mean scores for reported memory disjointedness and poor recall by group are displayed in Table 2. Mixed-measures ANOVAs revealed a significant main effect of time, *F*(1, 73) = 11.37, *p* = .001, η_*p*_^2^ = .14, 95% CI = [.02, .28], indicating that participants reported higher scores in disjointedness and poor recall at 1 week compared to Day 3. Furthermore, there was a significant Memory Quality × Group two-way interaction, *F*(1, 73) = 40.27, *p* < .001, η_*p*_^2^ = .36, 95% CI = [.18, .49] and a significant Time × Memory Quality two-way interaction, *F*(1, 73) = 6.61, *p* = .012, η_*p*_^2^ = .08, 95% CI = [.01, .22]. The Time × Memory Quality × Group three-way interaction was nonsignificant, *F*(1, 73) = 3.07, *p* = .08, η_*p*_^2^ = .04, 95% CI = [.00, .64]. The significant interactions were followed up with separate mixed-measures ANOVAs for poor recall and memory disjointedness. The ANOVA for memory disjointedness showed a significant main effect of group, *F*(1, 73) = 15.88, *p* < .001, η_*p*_^2^ = .18, 95% CI = [.05, .33], suggesting that the trauma-film group reported more memory disjointedness compared with the neutral-film group. There was no significant effect of time, *F*(1, 73) = 2.30, *p* = .13, η_*p*_^2^ = .03, 95% CI = [.00, .14], suggesting that memory disjointedness scores stayed stable with time in both groups. There was also no significant Time × Group interaction effect, *F*(1, 73) = 1.32, *p* < .26, η_*p*_^2^ = .02, 95% CI = [.00, .11]. The ANOVA for poor recall showed significant main effects of time, *F*(1, 73) = 13.62, *p* < .001, η_*p*_^2^ = .16, 95% CI = [.03, .30] and group, *F*(1, 73) = 4.00, *p* = .049, η_*p*_^2^ = .05, 95% CI = [.00, .17], indicating that poor memory recall increased with time and that the neutral-film group reported poorer recall compared with the trauma-film group. There was no Group × Time interaction effect, *F*(1, 73) = 0.90, *p* = .35, η_*p*_^2^ = .01, 95% CI = [.00, .10], indicating that the groups did not differ in how much poor recall increased with time.

### Hypothesis 3: trauma-memory qualities predict intrusions and PTSD-like symptoms

Correlations within the trauma-film group are displayed in Table 3. Poor memory recall showed very small correlations with the intrusion measures and IES-R scores, and only the correlations between poor recall and intrusions reported in the diary were significant. Memory disjointedness showed moderate to high correlations with intrusions in interview and diary at Day 3 and at 1-week follow-up and IES-R symptom scores, indicating that memory disjointedness at Day 3 correlated with the development of intrusions and PTSD-like symptoms and that the association persisted at 1-week follow-up. Peritraumatic data-driven processing and dissociation showed moderate to high correlations with intrusions reported in interview and diary, as well as IES-R scores. This indicates that participants who engaged in more data-driven processing and more dissociation while watching the trauma film were more likely to develop intrusions and PTSD-like symptoms about the trauma film in the week after the film.

**Table 3.** Correlations Between Trauma-Memory Quality at Day 3 and 1-Week Follow-Up, Peritraumatic Cognitive Processing, and Intrusions and PTSD-Like Symptoms in the Week Following Trauma-Film Exposure

	Intrusions		
Variable	Diary	Interview	IES-R
Trauma-memory quality			
Poor memory recall Day 3 1-week follow-up	.33* .26*	.24 .24	.18 .23
Memory disjointedness Day 3 1-week follow-up Cognitive processing Data-driven processing Dissociation	.44** .46** .37* .43**	.45** .49** .34* .42**	.56** .70** .31* .41**

Note: IES-R = Impact of Event Scale–Revised.

* *p* < .05. **p < .01.

### Hypothesis 4: trauma memory qualities mediate the relationship between peritraumatic processing and intrusions

#### Poor memory recall

Peritraumatic dissociation, *r*(50) = .34, *p* = .02, but not data-driven processing, *r*(50) = .20, *p* = .18, correlated with poor memory recall. As poor memory recall at Day 3 predicted intrusions reported in the diary, only this mediation analysis was calculated. The indirect effect for the relationship between peritraumatic dissociation and intrusions reported in the diary was nonsignificant, indirect effect = .06, *SE* = 06, CI = [–.01, .23], indicating that poor memory recall did not mediate the relationship between peritraumatic dissociation and intrusions reported in the diary.

#### Memory disjointedness

**Fig. 1.**
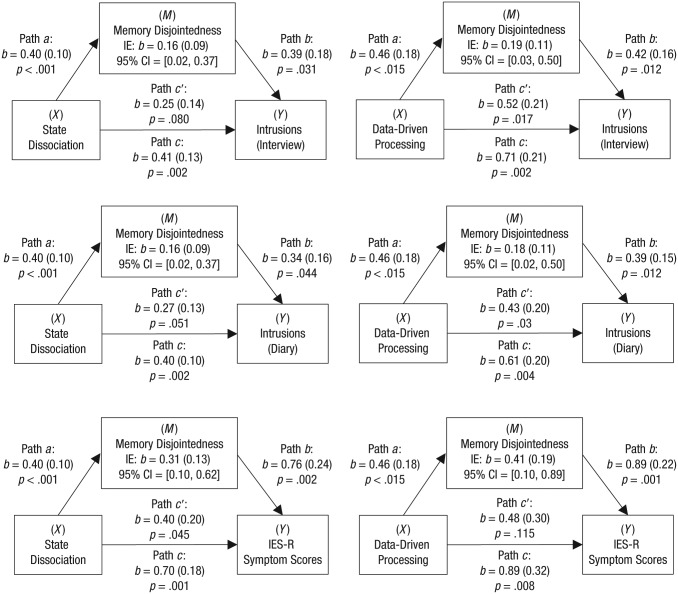
Mediation models showing the effect of independent variables (*X*) on dependent variables (*Y*), as mediated by memory disjointedness (*M*). Along the path from *X* to *Y* in each model, the value below the arrow (path *c*) shows the total effect, and the value above the arrow (path *c*′) shows the direct effect after controlling for *M*. The values in parentheses are standard errors. IE = indirect effect (path *a* × path *b*); CI = confidence interval (bias-corrected); IES-R = Impact of Event Scale-Revised.

Results of the mediation analyses for disjointedness are displayed in Figure 1. The indirect effects (*a* × *b*) for the relationship between peritraumatic cognitive processing and intrusions, as well as IES-R symptom scores via memory disjointedness, were significant. This indicates that memory disjointedness at least partially mediated the relationship between peritraumatic cognitive processing and PTSD symptoms at 1 week. The direct effect (*c*′) of dissociation on intrusions lost significance for intrusions reported in the diary and interview (full mediation) and was reduced but remained significant for IES-R symptom scores (partial mediation). The direct effect (*c*′) of data-driven processing on intrusions in the diary and interview was reduced but remained significant (partial mediation) and lost significance for IES-R symptom scores (full mediation). The predictors *memory disjointedness* and *dissociation* accounted for approximately 25% of the variance in intrusions reported in the diary (*R*^2^ = .26) and interview (*R*^2^ = .25) and for 38% (*R*^2^ = .38) of the variance in IES-R symptom scores. The predictors *memory disjointedness* and *data-driven processing* accounted for approximately 27% of the variance in intrusions reported in the diary (*R*^2^ = .27) and interview (*R*^2^ = .29) and 35% in the IES-R symptom scoress (*R*^2^ = .35).

## Discussion

In the present study we used a trauma-film paradigm to investigate the role of trauma exposure in the formation of disorganized memories and the role of memory disorganization in the development of intrusions. The study distinguished between poor recall (difficulties in remembering details of the trauma or the order of events) and memory disjointedness (poor links between the most upsetting moments and context information), as inconsistent results in the literature suggested that different aspects of memory disorganization may differ in their relevance to the development of re-experiencing symptoms. Film memories in the trauma-film group were characterized by greater disjointedness but not poorer recall than those in the neutral-film group. In the trauma-film group, memory disjointedness but not poor memory recall was strongly associated with the development of analogue PTSD symptoms. Memory disjointedness but not poor memory recall mediated the relationship between peritraumatic cognitive processing and the development of intrusions and PTSD-like symptoms in the trauma-film group.

In line with Hypothesis 1, the findings suggest that exposure to a trauma film led to greater memory disjointedness of the worst moments of the film, compared with neutral-film exposure. In contrast to Hypothesis 1 but in line with Hypothesis 2, trauma-film exposure led to less reported difficulty in memory recall for the film than exposure to a neutral film. Memory disjointedness remained stable over time in both groups, whereas poor memory recall increased with time in both groups. The findings are in line with both Berntsen et al.’s (2003) suggestion that trauma memories should be remembered better than neutral memories and Ehlers et al.’s (2004) suggestion that the disjointedness of the worst moments of the traumatic event from other relevant information in autobiographical memory contributes to the development of re-experiencing symptoms. The findings on memory disjointedness are in line with previous studies showing that the worst moments of the trauma are particularly disjointed or disorganized in PTSD (e.g., Evans et al., 2007; Kleim et al., 2008). The finding that the trauma film did not lead to more difficulties in memory recall than the neutral film appears to be in contrast with previous studies showing that people with PTSD had more difficulties in recalling the trauma memory than other negative events (e.g., Halligan et al., 2003; Jelinek et al., 2009). However, the latter studies used other negative events rather than neutral events as a comparator, so both events had a negative valence and were self-relevant. Neutral material that is not relevant to the self, such as the film clip used in the study, may be more easily forgotten than negative material.

In line with Hypothesis 3, memory disjointedness at Day 3 and 1-week follow-up was related to re-experiencing symptoms and PTSD-like symptoms in the week after trauma-film exposure. Difficulties in memory recall showed only small and mainly nonsignificant associations with intrusion and PTSD measures. This is in line with the suggestion that it is the disjointedness of the worst moments which is most relevant to the development of re-experiencing symptoms (Ehlers et al., 2004) rather than the overall quality of the recall of the trauma. It is also in line with a previous trauma-film study showing that difficulties in remembering the order of the film (one aspect of poor memory recall) were not associated with greater PTSD symptoms (Segovia, Strange, & Takarangi, 2016).

In line with Hypothesis 4, memory disjointedness fully mediated the effect of peritraumatic dissociation on the development of analogue re-experiencing symptoms and partially mediated the relationship between PTSD-like symptoms in the week after film exposure. Furthermore, memory disjointedness partially mediated the relationship between data-driven processing and analogue intrusions and fully mediated the relationship between data-driven processing and PTSD-like symptoms. This suggests that peritraumatic processing may influence how the trauma, and particularly its worst moments, is encoded, which in turn contributes to the development of PTSD symptoms. This is in line with current cognitive models of PTSD that suggest that peritraumatic processes may contribute to the nature of the trauma memory in PTSD, which in turn is thought to influence the development of persistent PTSD symptoms (e.g., Brewin, 2014; Ehlers & Clark, 2000). The fact that memory disjointedness only partially mediated some of the relationship between peritraumatic processing and PTSD analogue symptoms suggests that there are also other pathways to PTSD symptoms. More research is needed to better understand which cognitive processes contribute to which PTSD symptoms.

The study has several limitations. First, the study used an analogue trauma paradigm. Even though previous studies found that the trauma-film paradigm produces re-experiencing symptoms with a similar quality to trauma survivors with PTSD (e.g., Weidmann et al., 2009), it remains unclear whether processes during trauma-film exposure also correspond to those during real-life trauma experience. Second, the content of the trauma film may have influenced the results for memory recall. Participants were exposed to a scene without a complex story line, as most of the clip displayed the rape from one camera angle. It is conceivable that poor memory recall, such as difficulties in remembering the order of an event, may play a greater role when many different things happen in quick succession. Third, this study mainly used self-report measures. It would be interesting to investigate trauma memory characteristics with objective ratings of trauma narratives to better understand discrepancies in the current PTSD literature. Fourth, cognitive processes were assessed shortly after the film to avoid interference with the exposure. It cannot be ruled out that self-reported peritraumatic processing scores were, to an extent, influenced by posttrauma cognitions and the impact of the film. Finally, one may argue that the last item of the disjointedness scale could not only be interpreted as a lack of context when remembering these moments but also be understood by some as intrusive, even though the unintended quality of the memory is not mentioned. Further research is needed to determine this item. It correlated highly with the other disjointedness items and was therefore retained in the analysis.

In conclusion, the results shed some light on discrepant findings in the literature on trauma-memory disorganization. Whereas the results on poor memory recall support Berntsen et al.’s (2003) hypothesis that trauma memories are better remembered than neutral memories, the results on the disjointedness of the worst moment from context information support cognitive theories of PTSD that emphasize the role of the nature of the trauma memory in the development of PTSD symptoms (e.g., Brewin, 2014; Ehlers & Clark, 2000). Thus, the results suggest that a narrower definition of the critical features of trauma memories may help to better understand the development of re-experiencing symptoms in PTSD.
